# The Physicians' Visiting List for 1877

**Published:** 1877-02

**Authors:** 


*The Physicians Visiting List for* 1877.—Publishers: Lindsay and Blakiston, Philadelphia.

For years past we have been using this List as a Dental Appointment Book for which it is well adapted. Besides its convenience, it contains useful information on Hall's Ready Method. Poisons and their Antidotes, Almanac, &c., and is of such a size as to meet every requirement.

